# Genomic selection for resistance to mammalian bark stripping and associated chemical compounds in radiata pine

**DOI:** 10.1093/g3journal/jkac245

**Published:** 2022-10-11

**Authors:** Judith S Nantongo, Brad M Potts, Jaroslav Klápště, Natalie J Graham, Heidi S Dungey, Hugh Fitzgerald, Julianne M O'Reilly-Wapstra

**Affiliations:** School of Natural Sciences, University of Tasmania, Hobart, TAS 7001, Australia; National Agricultural Research Organization, P.O Box 1752, Mukono, Uganda; School of Natural Sciences, University of Tasmania, Hobart, TAS 7001, Australia; ARC Training Centre for Forest Value, Hobart, TAS 7001, Australia; Scion (New Zealand Forest Research Institute Ltd.), Rotorua 3046, New Zealand; Scion (New Zealand Forest Research Institute Ltd.), Rotorua 3046, New Zealand; Scion (New Zealand Forest Research Institute Ltd.), Rotorua 3046, New Zealand; School of Natural Sciences, University of Tasmania, Hobart, TAS 7001, Australia; School of Natural Sciences, University of Tasmania, Hobart, TAS 7001, Australia; ARC Training Centre for Forest Value, Hobart, TAS 7001, Australia

**Keywords:** genomics, chemistry, defense, bark stripping, *Pinus radiata*, Genomic Prediction, GenPred, Shared Data Resource

## Abstract

The integration of genomic data into genetic evaluations can facilitate the rapid selection of superior genotypes and accelerate the breeding cycle in trees. In this study, 390 trees from 74 control-pollinated families were genotyped using a 36K Axiom SNP array. A total of 15,624 high-quality SNPs were used to develop genomic prediction models for mammalian bark stripping, tree height, and selected primary and secondary chemical compounds in the bark. Genetic parameters from different genomic prediction methods—single-trait best linear unbiased prediction based on a marker-based relationship matrix (genomic best linear unbiased prediction), multitrait single-step genomic best linear unbiased prediction, which integrated the marker-based and pedigree-based relationship matrices (single-step genomic best linear unbiased prediction) and the single-trait generalized ridge regression—were compared to equivalent single- or multitrait pedigree-based approaches (ABLUP). The influence of the statistical distribution of data on the genetic parameters was assessed. Results indicated that the heritability estimates were increased nearly 2-fold with genomic models compared to the equivalent pedigree-based models. Predictive accuracy of the single-step genomic best linear unbiased prediction was higher than the ABLUP for most traits. Allowing for heterogeneity in marker effects through the use of generalized ridge regression did not markedly improve predictive ability over genomic best linear unbiased prediction, arguing that most of the chemical traits are modulated by many genes with small effects. Overall, the traits with low pedigree-based heritability benefited more from genomic models compared to the traits with high pedigree-based heritability. There was no evidence that data skewness or the presence of outliers affected the genomic or pedigree-based genetic estimates.

## Introduction

The implementation of genomic prediction in plants has offered new possibilities for maximizing genetic gains for economically important traits (Meuwissen *et al.* 2001; Crossa *et al.* 2017) and may enhance the efficiency of selecting herbivory-resistant genotypes. In conifers, breeding for resistance against pests and diseases has mainly relied on conventional phenotype-based methods (Carson 1989; Alfaro *et al.* 2004) and has been facilitated by quantitative genetic studies that investigate the genetic basis of the resistance mechanisms. Although results from these studies mostly indicate that resistance traits are under genetic control and can respond to selection, the often low narrow-sense heritability estimates for pest resistance (0.02–0.14) and the associated chemical traits (0.07–0.50) can reduce the precision of breeding value predictions of these traits (Moreira *et al.* 2013; Zas *et al.* 2017; Nantongo *et al.* 2020; Nantongo, Potts, Frickey, *et al.* 2021; Nantongo *et al.* 2021a). In addition, the inherently long generation intervals of trees and high phenotyping costs are always a challenge in tree breeding. Therefore, the potential improvement in prediction accuracy of breeding values for those traits with low heritability (Goddard 2009; Hayes *et al.* 2009; Iwata *et al.* 2011; Gamal El-Dien *et al.* 2016; Klápště *et al.* 2018; Stejskal *et al.* 2018; Suontama *et al.* 2018), coupled with the predicted reduction in the length of breeding cycles (Thistlethwaite *et al.* 2017; Klápště *et al.* 2018), should be a major motivation for incorporating genomic selection in breeding for resistance in conifers. However, the effectiveness of genomic prediction depends on the improvement in the accuracy of breeding value predictions.

The factors that affect the accuracy of breeding value predictions and, hence, the expected response to genomic-informed selection, such as the trait heritability, size of the training population, effective number of genomic fragments, and genetic relatedness between training and validation population have been well-documented (Desta and Ortiz 2014; Klápště *et al.* 2018; Momen *et al.* 2018; Stejskal *et al.* 2018). Additionally, the optimal choice of statistical methods for genomic estimated breeding value predictions differs with respect to the underlying genetic architecture. Methods, such as the genomic best linear unbiased prediction (GBLUP), single-step GBLUP (ssGBLUP), and ridge regression best linear unbiased prediction assume that all marker effects follow the same distribution and each marker explains a very small amount of variance (Meuwissen *et al.* 2001; Legarra *et al.* 2009; Misztal *et al.* 2013). In contrast, heterogeneity of marker effects is better accounted for in linear regularized (penalized) regression models, such as generalized ridge regression (GRR), least absolute shrinkage, and selection operator and elastic net, as well Bayesian methods like Bayes A/B/C/Cπ/R (Meuwissen *et al.* 2017). The ssGBLUP integrates all the phenotypic, pedigree, and genomic information available simultaneously to predict genomic breeding values for genotyped and nongenotyped individuals through the combined matrix *H* (Christensen and Lund 2010). This allows the use of all the available information in a genetic improvement program. Studies that have evaluated the relative predictive performance of the different approaches mostly indicate that the optimum approach is partly dependent on the genetic architecture and heritability of the trait involved (Meuwissen *et al.* 2001; Momen *et al.* 2018; Ogutu *et al.* 2012; Ratcliffe *et al.* 2017; Wang, Zhou, *et al.* 2018). Genetic architecture describes genotype–phenotype relationships for the loci contributing to phenotypic variation and includes the number of loci and their genomic location, number of alleles per locus, magnitude of their effects, patterns of pleiotropy, mode of gene action, and epigenetic effects (Momen *et al.* 2018). Complex quantitative traits that are controlled by many genes with small effects which is likely for resistance to herbivory (Kliebenstein 2014; Lenz *et al.* 2020), may be better predicted by methods that do not prioritize individual genetic markers (Desta and Ortiz 2014; Lenz *et al.* 2020). In conifers, however, studies also indicate that some herbivory resistance, as well as associated chemical resistance traits, may be controlled by genes with major effects (Porth *et al.* 2011). For such traits that are controlled by major genes, predictions and their accuracy can be favorably estimated by models that apply variable selection and differential shrinkage of allelic effects, such as GRR (Gianola *et al.* 2009; Resende *et al.* 2012). Additionally, for resistance which is mostly scored on qualitative or semiquantitative scales, and chemical data that are skewed (Burdon *et al.* 1992), regression models that support non-normal data may be more appropriate (Kärkkäinen and Sillanpää 2012). Therefore, for less studied traits, it is important to evaluate a broad range of statistical methods to identify those that can better model their genetic architecture. Accordingly, we tested the ability of 3 selected statistical methods—GBLUP, ssGBLUP, and GRR—to accurately estimate breeding values for (1) resistance of radiata pine (*Pinus radiata* D. Don) to marsupial bark stripping and (2) the bark chemical traits, some of which may impact susceptibility to herbivory*.* These methods were selected to represent alternative approaches and assumptions related to marker effects (Meuwissen *et al.* 2017; Wang, Xu, *et al.* 2018) and were compared to the relevant pedigree-based ABLUP. The advantage of multitrait over single-trait models in improving the accuracy of predictions especially for traits of low heritability has been demonstrated (Guo *et al.* 2014; Klápště, Dungey, Telfer, *et al.* 2020). Additionally, multitrait models account for the ability of multiple traits to evolve together. Multitrait models improve predictions by taking advantage of strong genetic correlations between traits (Calus and Veerkamp 2011), implying that little improvement in prediction will be detected in the presence of weak genetic correlations among traits.

In radiata pine, bark stripping (removal of bark from the stem), especially by the native marsupial Bennett’s wallaby (*Macropus rufogriseus*) that occurs between the ages of 1 and 6 years, has become the most important pest problem in Tasmania (Miller *et al.* 2014; Nantongo *et al.* 2020), affecting up to 40% of the plantations, with up to 80% of trees damaged in some plantations (Miller *et al.* 2014). Bark stripping can reduce the economic value of trees due to wood damage arising mainly because of frequent infections and spreading of the fungal pathogens in the stem. Nevertheless, this economic loss is one of several problems within these damaged stands. If more than 90% of stem circumference is stripped, the whole tree usually dies (Miller *et al.* 2014; Nantongo *et al.* 2020). Given that the financial costs of managing mammalian herbivores through fencing and culling in *P. radiata* plantations are high (Wildlife Management Branch 2011), there is interest in the potential for exploiting genetic variation in tree resistance to bark stripping to sustainably reduce damage. In radiata pine, selection for pest resistance has mostly been based on conventional approaches involving visual selection and trait screening over several pedigreed generations (Carson 1989; Dungey *et al.* 2009). Although there do not appear to be operational breeding programs focused on reducing the susceptibility of radiata pine to herbivory, various quantitative genetic studies have indicated the potential for selection against insects and mammalian bark damage and the associated chemical traits in various radiata pine populations (Moreira *et al.* 2013; Nantongo *et al.* 2020; Nantongo, Potts, Davies, *et al.* 2022; Nantongo *et al.* 2021a). However, estimates of the pedigree-based heritabilities (ranging from 0.07 to 0.50) and genetic correlations among marsupial bark stripping and associated chemical traits, which include terpenes, phenolics, and sugars, are usually low to moderate (Nantongo *et al.* 2020, 2021a), suggesting that these traits would possibly benefit from genomic selection. In conifer-herbivore systems, only one study has examined the potential benefits of genomic selection, in this case for white pine weevil resistance in Norway spruce (Lenz *et al.* 2020). To our knowledge, there is no study incorporating genomic selection for chemical compounds related to susceptibility or resistance mechanisms in breeding programs of conifers. For other economically important traits in radiata pine, studies have indicated potential genetic gain from using genomic selection (Whitehill *et al.* 2016; Li and Dungey 2018) and similar concepts could be adopted for resistance breeding. Currently, there are marker panels to identify known biotic threats such as *Dothistroma pini* (Li *et al.* 2015) and other genomic resources in radiata pine (Telfer *et al.* 2018, 2019) that can facilitate detailed genomic dissection of resistance and other traits of interest.

The present study aimed to:

1. compare heritability and predictive ability/accuracy of the GBLUP and pedigree-based (ABLUP) models for selected primary and secondary chemical compounds in the bark, including those associated with bark stripping in radiata pine. A key focus was examination of the potential improvement in heritability estimates of the bark chemical compounds and their additive genetic correlation with bark stripping and tree height with GBLUP;
2. assess the potential improvement in predictive ability/accuracy of the GBLUP through use of nonlinear GRR model; and
3. assess the contribution of additional genotyped data to the improvement in heritability, predictive ability/accuracy, and genetic correlations in integrated multitrait analyses (ssBLUP).

## Materials and methods

### Plant material

The genetic field trial at Wilmot in Tasmania, Australia (−41.454271° N, 146.106801° E, 580 m ASL) described in Nantongo, Potts, Davies, *et al.* (2022) was used for this study. Plant material was sourced from the New Zealand Radiata Pine Breeding Company. The trial comprised 74 control-pollinated (CP) full-sib and 1 open-pollinated families that were planted in rows and columns of an incomplete randomized block design of 26 replicates, each replicate comprising 3 incomplete blocks, with each family represented as a single tree plot within each replicate (therefore each replicate comprised 75 individuals, but only individuals from the 74 CP families were included in the study). The families represented 55 unique parents and 54 grandparents, with the number of families per parent ranging from 1 to 10 (average 2.6). Twenty replicates (1,372 trees) were accessible to marsupial browsing for assessment of bark stripping. Six replicates (390 trees) were protected by fencing from marsupial bark stripping from which samples for the chemical analysis and for genotyping were collected (Supplementary Fig. 1). These protected replicates were randomly scattered within the trial. Within the protected replicates, alternate trees had been subject to artificial bark stripping [details in Nantongo, Potts, Davies, *et al.* (2022) and Nantongo *et al.* (2020)]. The protected trees were subject to artificial and not marsupial bark stripping to control the size of the bark strip removed and the time of stripping in order to assess the chemical responses to stripping in a controlled manner. Trees in the protected replicates were genotyped and phenotyped for chemical traits, giving a maximum of 6 individual trees per family assayed. In total, across the whole trial 1,762 plants were alive at assessment age.

### Phenotypic data

#### Bark stripping

From the 20 unprotected replicates, bark stripping damage by marsupials was recorded on an individual-plant basis at 2 years of age (see Nantongo *et al.* 2020). The damage was scored on a categorical scale assigning zero (0) to nonstripped plants, 1 = <25% of the circumference stripped, 2 = 25–50%, 3 = 50–75%, 4 = >75–<100%, and 5 = 100% of the circumference stripped. Except for the scores 0 and 100, the remaining scores were converted to class mid-point values for the final analyses. At the same time, height was assessed in all 26 replicates. Since marsupial bark stripping was not uniform, spatial adjustment was made for bark stripping (see Nantongo *et al.* 2020). The other phenotypic traits studied were not spatially adjusted. To set the spatial term for bark stripping, every tree was uniquely identified by a row and column position within the trial, setting the absent, dead, and filler trees to missing values (Costa e Silva *et al.* 2001). The missing values were included as a fixed factor in the models (Dungey *et al.* 2014). The spatial term was then partitioned into spatially correlated (*ξ*) and uncorrelated (*η*) residuals. The spatially correlated error (*ξ*) was modeled using a first-order separable autoregressive model in the row and column directions (Butler *et al.* 2009; Dungey *et al.* 2013). However, in addition to the 2-dimensional separable first-order autoregressive spatial model, an independent residual (nugget-*ψI*_150_) was also added as a random term. Therefore, the residual structure *R* follows:

$$R=\sigma_{\xi }^{2}\left[\mathrm{AR}1\left(p_{\mathrm{col}}\right)⊗\mathrm{AR}1\left(p_{\mathrm{row}}\right)\right]+\sigma_{\eta }^{2}I,$$

where $\sigma_{\xi }^{2}$ is the spatially dependent residual variance and $\sigma_{\eta }^{2}$ is independent residual variance, ⊗ is the Kronecker product, and AR1(*p*) is a first-order autoregressive correlation matrix with autocorrelation *p* for columns (col) and rows (row) (Butler *et al.* 2009).

Three weeks after the marsupial bark stripping assessment was conducted, an experiment was initiated to assess the constitutive and induced chemical differences for all 74 families using trees in the 6 protected replicates (*n* = 390 plants due to some losses of trees or protection). Half of the plants were subject to artificial bark stripping (treated trees; *n* = 195) at time zero (T0) and half were untreated (*n* = 195) and used as controls [more details of the sampling are presented in Nantongo, Potts, Davies, *et al.* (2022)]. Briefly, alternate plants in the 6 replicates were systematically treated regardless of family. The tree at one corner of each replicate was selected as a control tree, the next one in the column was selected for treatment and this pattern was consistently followed across the 6 replicates of the trial. The artificial bark stripping treatment was applied by removing a vertical strip of 15 cm of bark (which included the inner and the outer bark), starting 2 cm above the ground, and covering 30% of the stem circumference. The dimensions were selected based on the most common browsing level observed in the field. Three weeks after treatment application, another bark strip was removed directly above the treatment site, for the chemical assessment. A strip was also removed from an equivalent position on the untreated plants in the protected replicates. From these 6 protected replicates, needle samples were also collected for chemistry and genetic analysis (Supplementary Fig. 1). Artificial bark stripping was not scored and was not included in the ‘bark stripping’ response variable of the genetic models, rather it was treated as binary fixed effect (see below).

#### Chemical analysis using near-infrared reflectance spectroscopy

The chemical data used in this study were the same as used in Nantongo *et al.* (2021a). The chemical compounds in the bark, i.e. terpenes, phenolics, fatty acids, sugars, and unknown compounds, were predicted for the 6 protected replicates by near-infrared spectroscopy (NIRS) according to the methods documented in Nantongo, Potts, Rodemann *et al.* (2021) and the associated wet chemistry methods documented in Nantongo, Potts, Davies, *et al.* (2022). In brief, chemical extractions were performed on 150 bark samples and NIRS prediction was used for the rest of the samples. Wet chemical extraction with dichloromethane (DCM) to target terpenes was carried out in 5 ml using 0.75 mg of fresh bark material. An acetone extraction was performed to target phenolics in 10 ml of 95% (v/v) aqueous acetone on 50 g of freeze-dried ground material. Sugars were extracted from 50 g of freeze-dried, ground material in 10 ml of hot water (Jones *et al.* 2002). The DCM extracts were analyzed by gas chromatography–mass spectrometry (GC-MS) while the acetone extracts and sugars were analyzed by ultra-high-performance liquid chromatography–mass spectrometry (UHPLC-MS). The procedures for the GC-MS and UHPLC-MS are detailed in Nantongo *et al.* (2021b). For the NIRS analysis, samples were scanned when fresh and when freeze-dried and ground according to the methods in Nantongo, Potts, Rodemann, *et al.* (2021a). However, only models based on freeze-dried and ground samples that gave better models were used. Near-infrared reflectance spectroscopy models were developed to predict the amounts of all chemical compounds quantified in the bark as indicated in Nantongo *et al.* (2021a). The stronger model of either the cross-validated or the externally validated model was used to predict the chemistry of the unknown samples. The amount of each of the 65 compounds listed in Nantongo *et al.* (2021a) was predicted by NIRS, but only 15 compounds in the bark predicted with models with *r*^2^ > 0.5 (Nantongo *et al.* 2021a) and which were well identified were selected for this study. Four of these compounds were shown to positively correlate with marsupial bark stripping in Nantongo *et al.* (2021a). The descriptive statistics and the statistical distribution of all traits considered in the study are shown in Table 1 and Supplementary Table 1.

**Table 1.** Descriptive statistics of bark stripping (*n* = 1,372), height (*n* = 1,762), and the chemical compounds (*n* = 390) used for estimating heritability, genetic correlation, and accuracy of genomic selection.

Id	Compound	Compound group	Min	Mean	Max	SD
	Bark stripping		0.00	25.20	100.00	33.20
	Height (cm)		77.00	163.70	257.00	30.40
1	α-Pinene	M	0.02	3.82	0.78	0.42
4	β-Pinene	M	−0.38	8.34	1.84	0.99
5	Camphene	M	0.00	0.03	0.01	0.00
6	Citronellal	M	−0.06	0.46	0.04	0.05
18	Trans-farnesol	SS	−0.02	0.10	0.02	0.02
20	Agathadiol	DG	−0.54	3.89	0.55	0.51
21	Agatholal	DG	−0.10	1.56	0.34	0.21
22	Copalol	DG	0.00	0.18	0.03	0.02
23	Levopimaral	DG	0.00	0.05	0.01	0.01
30	Dehydroabietic acid	DL	8.53	39.35	24.71	5.18
54	Fructose	S	−0.10	2.12	1.33	0.36
55	Glucose	S	0.31	3.06	1.53	0.43
56	Inositol	S	0.17	2.30	1.09	0.36
59	Linoleic acid	F	7.12	27.00	16.91	3.75
60	Linolenic acid	F	1.11	12.31	7.69	1.46

Chemical compounds were predicted from NIRS models developed from scanning freeze-dried and ground bark samples and only well-identified bark compounds with NIRS prediction models *r*^2^ > 0.5 were selected for this study (Nantongo *et al.* 2021a), in addition to marsupial bark stripping and tree height. The monoterpenoids (M), sesquiterpenoids (SS), and GC-MS diterpenoids (DG) compound groups are expressed as milligrams of heptadecane equivalents per gram of dry weight of the sample. The LC-MS diterpenoids (DL) and fatty acids (F) are expressed as milligrams of nonadecanoic equivalents per gram of dry weight. The sugars (S) are expressed in absolute amounts (Min = minimum, Max = maximum, SD = standard deviation). Each chemical compound was given a unique identifier (Id), which follows (Nantongo *et al.* 2021a) for ease of location in the tables.

### Genotyping

From the 6 protected replicates, needle samples were collected from all individuals (*n* = 390) and stored at −80°C before DNA extraction. Total genomic DNA was extracted using a commercial NucleoSpin Plant II kit (Machery-Nagel, Duren, Germany) with modifications (Telfer *et al.* 2013). DNA purity and concentration were evaluated using a NanoDrop 2000 spectrophotometer (Thermo Scientific, Waltham, MA, USA) and quantified using the Agilent 5200 fragment analyzer (Palo Alto, CA, USA). The samples were genotyped using the 36K axiom SNP chip for radiata pine (NZPRAD02) developed on the Axiom platform (Thermo Fisher Scientific, Waltham, MA, USA) (Graham *et al.* 2022). Currently, this is the densest SNP array for radiata pine, capable of assaying 36,285 SNPs. A total of 390 individuals were included in the final genotype data with a total of 27,000 SNPs. These genotype data were filtered to include only SNPs with a mean allele frequency >0.05 and maximum missing data of 0.4% using the rrBLUP package (Endelman 2011). This filtering resulted in the retention of 15,624 SNPs for analysis. The genotyping reproducibility rate was high (99.9%), as estimated from 10 samples that were replicated during DNA extraction.

### Statistical methods

Three selected genomic evaluation methods were compared—GBLUP, ssGBLUP and GRR. GBLUP and ssGBLUP do not estimate individual marker effects and the 2 methods involve different sample sizes. GBLUP and GRR used only the same sample set of trees that were genotyped and phenotyped (*n* = 390) and these were the plants in the 6 protected replicates for which chemistry data was available (Nantongo *et al.* 2021a). The ssGBLUP included all individuals in the trial with documented pedigree that had been phenotyped for height (26 replicates, *n* = 1,372). Of these, 20 replicates were exposed to marsupial bark stripping and 6 replicates were protected (see above). The genomic models were compared to the pedigree-based (ABLUP) model involving the same individuals, which is the standard method used for breeding value prediction using the expected relatedness among individuals based on pedigree information.

The ssGBLUP and GBLUP models are the same as the ABLUP models detailed in Nantongo *et al.* (2021a), except that the average numerator relationship matrix ***A*** in the ABLUP is substituted with the realized genomic relationship matrix (***G***) in GBLUP and with the ***H***-matrix that combines ***G*** and ***A*** matrices in ssGBLUP (Christensen and Lund 2010).

The ***G***-matrix was computed using the “A.mat” function in the R package “rrBLUP” (Endelman 2011) from the marker data following (VanRaden 2008):

(1) G=ZZ'2∑ipi(1-pi),

where ***Z ***=*** M ***−*** P***, *M* is the matrix of genotypes coded 0, 1, and 2 as reference allele homozygote, heterozygote, and alternative allele homozygote, respectively, and ***P*** is the matrix of doubled frequencies for alternative alleles, *p_i_* is the frequency of the alternative allele at the *i*th locus (Christensen and Lund 2010).

The ssGBLUP combines the pedigree relationship matrix ***A*** and the genomic relationship matrix ***G***, in one matrix, ***H*** and hence simultaneously uses information from genotyped and nongenotyped individuals. The ***H***-matrix is defined by

(2) $$H\;=\;\left[\begin{array}{cc} A_{11}+A_{12}A_{22}^{-1}(G_{s}-A_{22})A_{22}^{-1}A_{21} & A_{12}A_{22}^{-1}G_{s}\\ G_{s}A_{22}^{-1}A_{21} & G_{s} \end{array}\right],$$

where ***A*_11_** represents the relationship matrix for the nongenotyped individuals (20 replicates, *n* = 1,372), ***A*_12_** and ***A*_21_** are relationship matrices between genotyped and nongenotyped individuals (26 replicates, *n* = 1,762), while ***A*_22_** is the pedigree-based relationship matrix for genotyped individuals (6 replicates, *n* = 390) and ***G_s_*** is the scaled marker-based ***G***-matrix for only the genotyped individuals (see scaling below). ***A*_22_^−1^** is the inverse of ***A*_22_**.

Forming the ***H***-matrix above involves 2-major steps. First, a matrix ***G_a_*** is created from ***G*** such that the average of its diagonal elements (avg.diag) and average of the nondiagonal elements (avg.offdiag) is equal to the average of the diagonal and off-diagonal elements of ***A*_22_**, respectively. Following (Gao *et al.* 2012), this was done by applying adjustment factors, *α* and *β*, to all elements of ***G***:

(3) $$G_{a}=G\beta +\alpha ,$$

where *α* and *β* are adjustment factors derived from the following simultaneous equations:

(4) $$\{\begin{array}{c} \text{Avg}.\text{diag}\;\left(G\right)\;\beta +\alpha =\text{Avg}.\text{diag}\left(A_{22}\right)\\ \text{Avg}.\text{offdiag}\;\left(G\right)\;\beta +\alpha =\text{Avg}.\text{offdiag}\left(A_{22}\right) \end{array}.$$

The ***G*** matrix is usually not positive semi-definite, which is one of the mixed linear model assumptions, and weighting of the genomic and pedigree-based relationship matrices is required as follows:

(5) $$G_{s}=G_{a}\left(1-w\right)\;+A_{22}w,$$

where ***G_s_*** is a rescaled genomic relationship matrix based on the SNP data, ***G_a_*** is the adjusted genomic relationship matrix [Equation (3)] and *w* is the weighting factor that represents the fraction of total additive variance that is not captured by markers and ***A*_22_** is the additive relationship matrix from the full pedigree. The weight (*w*) can take any value between 0 and 1, where the model with *w* = 1 is equivalent to ABLUP. For the present study, an arbitrary *w* of 0.05 was selected to give high weighting to the genomic data (Martini *et al.* 2018).

## Linear models for estimation of variance components

Variance components based on the single-trait pedigree-based relationship matrix (***A***) have been previously documented (Nantongo *et al.* 2021a). This study presents the results from single- and multitrait ABLUP for comparison with the genomic models. The variance components for the ABLUP, ssGBLUP, or GBLUP were obtained in ASReml-R v4.1 (Gilmour *et al.* 2015, R Core Team, 2013) using a general linear additive genetic model as

(6) $$y=X\beta +Z_{1}a+Z_{2}b\;+\;Z_{3}r\;+e,$$

where ***y*** is the response variable (height, spatially adjusted marsupial bark stripping, and a chemical variable); ***β***, a vector of fixed effects. For single-trait models, the fixed term ***β*** contained the overall phenotypic mean and the treatment term was fitted for the chemical traits. In addition, in the multitrait models another fixed term, “protected” was fitted for height to differentiate the measurements from the 20 replicates that were unprotected—(where marsupial bark stripping was scored)—from those from the 6 protected replicates from which chemistry was estimated. The model term, ***a*** is the vector of random additive effects following ∼*N* (0, ***A*** *σ_a_*^2^), *b* is the vector of random incomplete block effects following ∼*N* (0, ***I****σ_b_*^2^), *r* is the vector of random replicate effects following ∼*N* (0, ***I****σ_r_*^2^), and ***e*** is a vector of random residuals following ∼*N* (0, ***I****σ_e_*^2^). The additive genetic variance (*σ_a_*^2^) is based on the ***A***, ***G***, or ***H*** relationship matrices. The random family (specific combining ability) and the ***a*** x treatment terms were excluded from the analyses because they were generally nonsignificant in previous analyses (Nantongo *et al.* 2021a). ***X*** and ***Z***_1–3_ correspond to design matrices relating the observations in ***y*** to the fixed and random effects, respectively. To test whether the additive genetic variation was greater than zero, full models were compared with respective reduced models using a 1-tailed likelihood ratio test (LRT) (Gilmour *et al.* 2015) for all the GBLUP and ssGBLUP models.

Narrow-sense heritability estimates were derived from single-trait ABLUP and GBLUP as well as multitrait ABLUP and multitrait ssGBLUP models (see below for multitrait models). Individual narrow-sense heritability ($\hat{h}$^2^) was estimated as the additive genetic variance divided by the sum of the additive genetic variance ${\hat{\sigma }}_{a}^{2}\;$and the error variance ${\hat{\sigma }}_{e}^{2}$ as below:

(7) $${\hat{h}}^{2}=\frac{{\hat{\sigma }}_{a}^{2}}{{\hat{\sigma }}_{a}^{2}+{\hat{\sigma }}_{e}^{2}}.$$

Estimates of the associated standard error (SE) for the traits were obtained directly using Taylor series expansion (“delta method”) (Gilmour *et al.* 2015). The experimental fixed effects (i.e. treatment, protection) and design variances were excluded from the denominator of the heritability equation, as in previous analyses (Nantongo *et al.* 2021a) and thus the estimates are conditional on these factors. To test whether the additive genetic variation was greater than zero, full models were compared with respective reduced models using 1-tailed log LRTs with 1 degree of freedom in ASReml (Butler *et al.* 2009). The single-trait ABLUP heritability values presented were those from Nantongo *et al.* (2021a). The heritability estimates and SE reported for height and marsupial bark stripping of multitrait models are average values obtained from the 15 models.

Comparisons of the heritability estimated were made between pedigree-based and marker-based models. The single-trait GBLUP estimates were compared to single-trait ABLUP estimates calculated using only the genotyped individuals (*n* = 390). The multitrait ssGBLUP estimates were compared with the multitrait ABLUP estimates based on genotyped individuals plus those which had been measured for height (*n* = 1,372). A 2-tailed paired *t*-test was used to test the average difference in the heritability estimates between the different types of analyses (e.g. ABLUP vs GBLUP) using estimates from the 15 bark chemicals. The additive genetic estimates for the 15 chemical compounds from the single-trait pedigree-based model and the same set of individuals as used in the GBLUP and GRR analyses have been previously published (Nantongo *et al.* 2021a) and are presented here for comparative purposes.

## Genetic correlations

The multitrait models were also used to study the improvement of estimates of the genetic correlations of the chemical traits with spatially adjusted marsupial bark stripping and height. The multitrait models were as described above which were fitted for height, spatially adjusted marsupial bark stripping, and one chemical compound as response variables using ABLUP and ssGBLUP. The genetic correlation (*r_g_*) between 2 traits measured was estimated as:

(8) $$r_{g}=\frac{\sigma_{\mathrm{axay}}}{\sqrt{\sigma_{ax}^{2}\cdot \sigma_{ay}^{2}}},$$

where *σ_axay_* is the additive genetic covariance between traits *x* and *y*, $\sigma^{2}{}_{ax}$ is the additive genetic variance components for trait *x*, and $\sigma^{2}{}_{ay}$ is the additive genetic variance components for trait *y*. SEs were estimated in ASReml-R (Gilmour *et al.* 2015, R Core Team, 2013). To test whether genetic correlations were significantly different from zero, a full model was compared with the respective reduced model that had the additive covariances fixed to zero using 2-sided LRT with 1 degree of freedom in ASReml-R. The average difference in the genetic correlation estimates from ssGBLUP and those obtained with the multitrait ABLUP (Nantongo *et al.* 2021a) was tested using a 2-tailed paired *t*-test. The difference in the SEs of the genetic correlations was similarly tested, as a test of the difference in the accuracy of these estimates.

## Ridge regression

Ridge regression is one of a family of penalized regression methods that was originally proposed as a means of estimating regression coefficients with smaller mean-square error than their least squares counterparts when predictors are correlated (Frouin *et al.* 2020). Ridge regression also uses a general linear equation;

(9) $$y=µ+Z_{1}g+Z_{2}g+\varepsilon ,$$

where ***y*** is a (*n* × 1) vector of a response variable (height, spatially adjusted marsupial bark stripping, and a chemical variable) and *µ* is the phenotypic mean. The variable ***g*** is the vector of random effects, ***Z*_1_** and ***Z*_2_** are (*n* × *p*) design matrices of rank *p* for SNP and design effects respectively and ***ε*** is a (*n* × 1) vector of random residuals that are respectively assumed to follow a normal distribution, i.e. ***g*** ∼ *N* (0, *Iσ_g_*^2^) and***e*** ∼ *N* (0, *Iσ_e_*^2^), where *I* is an identity matrix.

Generalized ridge regression (GRR) estimates marker effects using linear and penalized parameters. It alters the notations of parameter *b* in Equation (8) by allowing variable shrinkage for different markers through the introduction of a diagonal matrix following a 2-step process (Shen *et al.* 2013).

In the first step, the predicted breeding values are obtained following the mixed model by the summing of all the marker effects of an individual tree [Equation (8)]. The solution for the marker effects is given by the following equation:

(10) $$\hat{g}={\left(z'z+\lambda I\right)}^{-1}z'y,$$

where $\lambda =\sigma_{e}^{2}/\sigma_{g}^{2}$, and is the ridge penalty parameter (Shen *et al.* 2013; Veerman *et al.* 2022).

In the second step, the BLUPs $\hat{g}$ are re-estimated using Equation (9) but with a marker-specific shrinkage parameters diag (*λ_i_*) instead of *λ****I***, where each *λ_i_* depends on the value $\hat{gi}$ from the first step. The equation now becomes

$$\hat{g}={\left(z'z+\mathrm{diag}\left(\lambda \right)\right)}^{-1}z'y,$$

where λ is a vector of shrinkage parameters and $\hat{g}$ is the BLUP marker effect (from step 1) (Rönnegård and Shen 2016). GRR was implemented in the “bigRR” package in R (Rönnegård and Shen 2016) using only the genotyped individuals (*n* = 390).

Marker effects for GBLUP and GRR were generated in R using rrBLUP (Endelman 2011). Design effects were derived from the experimental blocks and replicates. Treatment effects were not included here since they were not significant in previous studies (Nantongo *et al.* 2021a).

## Cross-validation scheme for estimating predictive accuracy

The predictive ability and predictive accuracy assess the potential of the models to estimate the breeding value of individuals with yet-to-be observed phenotypes (Momen *et al.* 2018). To test the predictive ability and accuracy of the 3 genomic and ABLUP methods, a 10-fold cross-validation scheme was implemented in ASReml-R (Utz *et al.* 2000; Gianola and Schön 2016). The model terms were the same as those used in the previous analyses. Within each model, all phenotyped and nonphenotyped individuals were randomly subdivided into 10 subsets (i.e. folds), and a leave one out procedure was repeated 10 times until all individuals had their breeding values predicted. Predictive ability of phenotypes was then defined as the Pearson correlation between the genomic estimated breeding values predicted in cross-validation and the observed phenotypes. Predictive accuracy was calculated by dividing the predictive ability by the square root of the single-trait ABLUP heritability (Momen *et al.* 2018). A 2-tailed paired *t*-test was used to test the average difference in the prediction estimates as above. The effect of data distribution on predictive ability was also assessed as indicated in the Supplementary methods (Supplementary File 1).

## Results

### ABLUP vs GBLUP

#### Additive genetic variance and heritabilities

The single-trait ABLUP and GBLUP models employed the same number of individuals and random as well as fixed effects and are directly comparable. The ABLUP narrow-sense heritability values of the 15 chemical compounds ranged between 0.12 and 0.51 and averaged 0.27 ± 0.10 (Table 2). Based on the GBLUP, heritability estimates for the 15 chemical traits studied ranged from 0.18 to 0.57 and averaged 0.43 ± 0.11. This average was 1.6-fold higher than the average of the single-trait ABLUP heritability estimates based on the same model and set of individuals (Table 2) and represented a significant increase in heritability estimates by the use of the *G* matrix rather than the pedigree-derived *A* matrix (paired *t*_14_ = 7.18, *P* < 0.001). The highest proportional increase was exhibited for compounds that had the lowest single-trait ABLUP heritability. For example, the heritability of citronellal^[6]^, trans-farnesol^[18]^, agathadiol^[20]^, and inositol^[56]^ increased between 2.1- and 2.7-fold with the GBLUP. Apart from the heritability of linoleic acid^[59]^ that slightly reduced, the compounds experienced between 1.10- and 2.09-fold increases in heritability with GBLUP compared to ABLUP. Significant additive genetic variation was also detected for agathadiol^[20]^, dehydroabietic acid^[30]^, and inositol^[56]^ that were not significant with the ABLUP models. Regardless of the set of samples or the type of analysis, the heritability estimates for bark stripping and tree height were low. For example, the height heritability of the ABLUP (*h*^2^ = 0.05 ± 0.02) based on the 20 unprotected replicates as well as the GBLUP heritability (*h*^2^ = 0.09 ± 0.07) based on 6 protected replicates were low. Based on unprotected trees, significant additive genetic variation but low heritability was exhibited for the spatially adjusted marsupial bark stripping (*h*^2^ = 0.09 ± 0.03) (Table 2).

**Table 2.** Narrow-sense heritability (*h*^2^) and standard error (SE) estimates of selected chemical compounds quantified in *P. radiata* bark based on single-trait models for ABLUP and GBLUP (protected replicates only, *n* = 390) and multitrait ABLUP and ssGBLUP (protected and unprotected replicates, *n* = 1,372).

			Single trait				Multitrait			
Id	Compound	Group	ABLUP heritability (SE)		GBLUP heritability (SE)		ABLUP heritability (SE)		ssGBLUP heritability (SE)	
	Bark stripping		0.09	(0.03)^b^			0.04	(0.02)	0.12	(0.04)
	Height		0.05	(0.02)^a^	0.09	(0.07)^a^	0.05	(0.02)	0.07	(0.03)
1	α-Pinene	M	0.26	(0.10)^c^	0.33	(0.11)^c^	0.25	(0.10)	0.33	(0.11)
4	β-Pinene	M	0.33	(0.11)^c^	0.57	(0.11)^c^	0.34	(0.12)	0.60	(0.11)
5	Camphene	M	0.30	(0.10)^c^	0.52	(0.11)^c^	0.29	(0.11)	0.55	(0.11)
6	Citronellal	M	0.19	(0.09)^c^	0.41	(0.13)^c^	0.19	(0.09)	0.49	(0.11)
18	Trans-farnesol	SS	0.15	(0.09)^c^	0.34	(0.12)^c^	0.14	(0.08)	0.44	(0.12)
20	Agathadiol	DG	0.22	(0.10)^c^	0.46	(0.12)^c^	0.22	(0.10)	0.46	(0.12)
21	Agatholal	DG	0.22	(0.10)	0.34	(0.11)^c^	0.24	(0.10)	0.35	(0.11)
22	Copalol	DG	0.29	(0.10)^c^	0.51	(0.11)^c^	0.28	(0.10)	0.52	(0.11)
23	Levopimaral	DG	0.31	(0.11)^c^	0.51	(0.11)^c^	0.32	(0.11)	0.54	(0.11)
30	Dehydroabietic acid	DL	0.12	(0.07)	0.18	(0.09)^a^	0.10	(0.07)	0.18	(0.09)
54	Fructose	S	0.31	(0.11)^b^	0.48	(0.11)^c^	0.30	(0.10)	0.47	(0.10)
55	Glucose	S	0.29	(0.11)^b^	0.51	(0.10)^c^	0.27	(0.10)	0.49	(0.10)
56	Inositol	S	0.15	(0.08)	0.32	(0.11)^c^	0.16	(0.09)	0.33	(0.11)
59	Linoleic acid	F	0.51	(0.14)^c^	0.48	(0.10)^c^	0.55	(0.13)	0.46	(0.10)
60	Linolenic acid	F	0.44	(0.13)^c^	0.48	(0.10)^c^	0.45	(0.12)	0.47	(0.10)

The single-trait ABLUP heritability estimates of marsupial bark stripping and tree height were derived from the 20 unprotected replicates. The GBLUP heritability for height is, however, based on the 6 protected replicates. The multitrait models included height, spatially adjusted marsupial bark stripping, and one chemical compound. The significance that the additive genetic variation from the single-trait ABLUP and GBLUP was greater than zero was tested using the 1-tailed likelihood ratio test (Nantongo *et al.* 2021a), where

a *P* < 0.05,

b *P* < 0.01, and

c *P* < 0.001. Significance tests were not done for the multitrait models. M = monoterpenoids, S = sesquiterpenoids, DG = GC-MS diterpenoids, DL = LC-MS diterpenoids, S = sugars and F = fatty acids. Each chemical compound is given a unique identifier (Id) for ease of location in the tables. The heritability estimates and standard error (SE) reported for height and marsupial bark stripping from multitrait models is an average of heritability values obtained from the 15 models. The ABLUP additive genetic and heritability estimates for the 15 chemical compounds from the single-trait pedigree-based model are based on the same set of individuals as used in the GBLUP and GRR analyses and have been previously published (Nantongo *et al.* 2021a) and are presented here for comparative purposes.

#### Predictive ability/accuracy of ABLUP, GBLUP, and GRR

The predictive ability (Pab) and predictive accuracy (Pac) for the height and the bark chemical compounds varied between −0.02 to 0.40 and −0.06 to 0.77, respectively, depending on the trait and statistical model (Table 3). Comparing single-trait models for chemical traits, the predictive ability for single-trait ABLUP (average Pab = 0.24) did not differ significantly from GBLUP (average Pab = 0.24) (paired *t*_14_ = 0.17, *P* = 0.43) and the GRR (average Pab = 0.23, paired *t*_14_ = 0.41, *P* = 0.34). Accounting for marker heterogeneity has little influence on Pab/Pac, although marker effects changed up to 10-fold in GRR compared to GBLUP (Supplementary Fig. 2). For example, the Pab of GBLUP did not differ from that of GRR (paired *t*_14_ = 0.37, *P* = 0.36). Similarly, the predictive accuracy did not differ between the ABLUP (average Pac = 0.47) and GBLUP (average Pac = 0.45) (paired *t*_14_ = 0.37, *P* = 0.36), ABLUP and GRR (average Pac =0.44) (paired *t*_14_ = 0.61, *P* = 0.27), nor between GBLUP and GRR (paired *t*_14_ = 0.36, *P* = 0.36).

**Table 3.** The predictive ability (Pab) defined as the correlation of breeding values from 10-fold cross-validation with observed phenotypic values and predictive accuracy (Pac) of the bark chemical compounds.

			Predictive ability						Predictive accuracy				
			Single-trait			Multitrait			Single-trait			Multitrait	
Id	Trait	Group	ABLUP	GBLUP	GRR	ABLUP	ssGBLUP		ABLUP	GBLUP	GRR	ABLUP	ssGBLUP
	Bark stripping					0.16	0.17					0.62	0.65
	Height					0.09	0.09					0.47	0.46
1	α-Pinene	M	0.23	0.22	0.22	0.25	0.25		0.46	0.44	0.42	0.49	0.49
4	β-Pinene	M	0.27	0.31	0.30	0.26	0.37		0.46	0.54	0.52	0.46	0.64
5	Camphene	M	0.24	0.36	0.28	0.22	0.34		0.44	0.66	0.50	0.40	0.63
6	Citronellal	M	0.25	0.30	0.29	0.24	0.33		0.58	0.69	0.66	0.54	0.77
18	Trans-farnesol	SS	0.23	0.20	0.24	0.11	0.28		0.59	0.51	0.62	0.29	0.71
20	Agathadiol	DG	0.27	0.24	0.27	0.16	0.26		0.58	0.50	0.57	0.35	0.55
21	Agatholal	DG	0.21	0.22	0.23	0.19	0.26		0.46	0.46	0.49	0.40	0.56
22	Copalol	DG	0.29	0.36	0.35	0.30	0.40		0.53	0.67	0.66	0.55	0.74
23	Levopimaral	DG	0.40	0.33	0.30	0.30	0.35		0.72	0.59	0.55	0.55	0.63
30	Dehydroabietic acid	DL	0.02	−0.02	−0.11	0.07	0.08		0.07	−0.06	−0.32	0.21	0.24
54	Fructose	S	0.20	0.26	0.27	0.22	0.32		0.35	0.47	0.49	0.39	0.57
55	Glucose	S	0.17	0.25	0.26	0.19	0.36		0.31	0.46	0.48	0.36	0.66
56	Inositol	S	0.25	0.04	0.11	0.12	0.20		0.64	0.11	0.27	0.31	0.52
59	Linoleic acid	F	0.32	0.17	0.20	0.33	0.31		0.45	0.24	0.28	0.46	0.44
60	Linolenic acid	F	0.27	0.32	0.29	0.33	0.40		0.40	0.49	0.44	0.50	0.60

Each chemical compound is given a unique identifier (Id) for ease of location in the tables. The single-trait analyses are based on the protected replicates (*n* = 390) and the multitrait analyses include the protected and unprotected replicates (*n* = 1,372) of the field trial. The predictive accuracy is calculated as the predictive ability divided by the square root of single-trait ABLUP heritability (i.e. Pac = Pab/h). M = monoterpenoids, S = sesquiterpenoids, DG = GC-MS diterpenoids, DL = LC-MS diterpenoids, S = sugars and F = fatty acids. Each chemical compound is given a unique identifier (Id) for ease of location in the tables.

### Multitrait ABLUP vs ssGBLUP—incorporating genotyped and ungenotyped individuals

#### Additive genetic variance and heritabilities

The heritability estimates for the 15 bark chemical compounds were similarly improved with the incorporation of genomic-derived pedigree information and additional phenotyped individuals through the use of the *H* matrix in the multitrait ssGBLUP analysis compared with estimates from the equivalent ABLUP analysis which incorporates genotyped (6 replicates) and un-genotyped trees (20 replicates) (Table 2). The multitrait ssGBLUP heritability estimates for individual bark compounds ranged between 0.18 and 0.61 and averaged 0.45 ± 0.10. As with the single-trait analyses, these estimates were significantly (paired *t*_14_ = 5.96, *P* < 0.001), and on average 1.5-fold, greater than the heritabilities estimated from multitrait ABLUP (average = 0.27 ± 0.10). In the multitrait analysis of height involving all individuals in the trial, the improvement in heritability was increased 1.4 times (ABLUP *h*^2^ = 0.05 ± 0.02, ssGBLUP *h*^2^ = 0.07 ± 0.03). However, for bark stripping the multitrait ssGBLUP heritability estimate (*h*^2^ = 0.12 ± 0.04) was 3 times higher than the ABLUP estimate (*h*^2^ = 0.04 ± 0.02). The multitrait ABLUP heritability estimates for marsupial bark stripping and height were relatively constant irrespective of the chemical compound fitted in the model (data not shown).

#### Predictive ability/accuracy of multitrait ABLUP and ssGBLUP

For bark chemicals, the predictive ability for the multitrait models increased significantly from an average of 0.22 with multitrait ABLUP to 0.30 with ssGBLUP (paired *t*_14_ = 5.72, *P* < 0.001). The predictive accuracy similarly increased from an average of 0.41 to 0.69 (*t*_14_ = 5.56, *P* < 0.001). The linear relationship between heritability and predictive ability for the 15 chemical compounds was high, indicating that there was a tendency for compounds with higher heritability estimates to reach higher predictive ability for all models (Supplementary Fig. 3). There was no significant linear relationship between heritability and Pac (results not shown), as this was in part corrected for heritability (by definition).

##### Genetic correlations

The genetic correlations of chemical compounds with marsupial bark stripping or height varied from positive to negative for both multitrait ABLUP and ssGBLUP (Table 4). Although most correlations retained the same sign across analyses, shifts from the low negative (ABLUP) to low positive (ssGBLUP) genetic correlations were common. The absolute values of positive correlations detected with ABLUP marginally increased with ssGBLUP (Table 4). The ABLUP negative correlations either changed to positive or reduced in magnitude with the ssGBLUP. Overall, the average of the genetic correlations between chemical compounds with bark stripping increased with ssGBLUP (*r_g_* = 0.21) compared with ABLUP (*r_g_* = 0.11, paired *t*_14_ = 3.09, *P* < 0.01). Similarly, genetic correlations between chemical compounds with height increased with ssGBLUP (*r_g_* = 0.19) compared with ABLUP (*r_g_* = 0.10, paired *t*_14_ = 2.66, *P* < 0.01). Further, the SE of the genetic correlation estimates obtained with ssGBLUP were on average significantly lower than those obtained with ABLUP for both marsupial bark stripping (ABLUP average SE = 0.28, ssGBLUP average SE = 0.25, paired *t*_14_ = 3.38, *P* < 0.01) and height (ABLUP average SE = 0.32, ssGBLUP average SE = 0.29, paired *t*_14_ = 5.86, *P* < 0.001), suggesting slightly more accurate estimates with the ssGBLUP model. The ABLUP correlation estimates that were associated with the highest SEs more strongly increased in accuracy than those that had low SEs of estimation (Table 4). The genetic correlation between height and marsupial bark stripping did not change with ssGBLUP (av. *r_g_* ± av. SE = 0.41 ± 0.27) compared to ABLUP (av. *r_g_* ± av. SE = 0.41 ± 0.29) although the correlation varied depending on the chemical compound in the model (Supplementary Table 2).

**Table 4.** Additive genetic correlation of different bark chemical compounds with marsupial bark stripping and height estimated using the pedigree-based method (ABLUP) and single-step GBLUP (ssGBLUP) multitrait models (protected and unprotected replicates, *n* = 1,372).

		Genetic correlation with bark stripping (*r_g_*) (SE)		Genetic correlation with height (*r_g_*) (SE)	
Id	Compound	ABLUP	ssGBLUP	ABLUP	ssGBLUP
**1**	α-Pinene	−0.20 (0.29)	−0.01 (0.28)	0.02 (0.33)	0.15 (0.30)
**4**	β-Pinene	−0.01 (0.28)	0.14 (0.25)	−0.04 (0.32)	0.15 (0.26)
**5**	Camphene	−0.12 (0.28)	0.01 (0.26)	−0.00 (0.33)	0.07 (0.27)
**6**	Citronellal	−0.14 (0.32)	−0.17 (0.27)	−0.32 (0.36)	−0.16 (0.29)
**18**	Trans-farnesol	0.08 (0.35)	0.25 (0.26)	−0.37 (0.39)	0.01 (0.29)
**20**	Agathadiol	−0.03 (0.31)	0.14 (0.27)	0.00 (0.36)	0.18 (0.29)
**21**	Agatholal	−0.12 (0.29)	0.07 (0.27)	0.18 (0.33)	0.24 (0.29)
**22**	Copalol	−0.09 (0.28)	0.05 (0.26)	−0.20 (0.32)	−0.02 (0.28)
**23**	Levopimaral	0.08 (0.27)	0.16 (0.25)	0.04 (0.31)	0.11 (0.27)
**30**	Dehydroabietic acid	−0.27 (0.37)	0.10 (0.33)	0.39 (0.39)	0.43 (0.32)
**54**	Fructose	0.55 (0.23)^a^	0.51 (0.22)^a^	0.06 (0.31)	0.08 (0.27)
**55**	Glucose	0.80 (0.20)^b^	0.71 (0.19)^b^	0.62 (0.24)^b^	0.52 (0.23)^a^
**56**	Inositol	−0.14 (0.33)	−0.04 (0.29)	−0.01 (0.38)	−0.01 (0.32)
**59**	Linoleic acid	0.68 (0.16)^b^	0.65 (0.19)^b^	0.69 (0.22)^a^	0.61 (0.24)^a^
**60**	Linolenic acid	0.65 (0.19)^b^	0.62 (0.20)^a^	0.50 (0.26)^b^	0.47 (0.25)^a^

The multitrait models always included the spatially adjusted marsupial bark-stripping scores, tree height, and one of the listed bark chemical compounds as response variables. The significance that the additive genetic correlation (*r_g_*) is different from zero was tested using a 2-tailed likelihood ratio test (Nantongo *et al.* 2021a) and is presented here for comparative purposes.

a *P* < 0.05,

b *P* < 0.01.

No new insights were obtained in terms of the chemical compounds associated with bark stripping. Importantly, the genetic correlations of the sugars—glucose^[55]^ and fructose^[54]^—as well as the fatty acids—linoleic acid^[59]^ and linolenic acid^[60]^—with marsupial bark stripping that were significant with the multitrait ABLUP model were still significant with the multitrait ssGBLUP, although the magnitude of the genetic correlations slightly reduced (Table 4). The positive genetic correlations of 3 compounds with height that were significant with the ABLUP models were slightly reduced with ssGBLUP, but 2 of the 3 compounds still retained a statistically significant genetic correlation (Table 4).

**Fig. 1.**
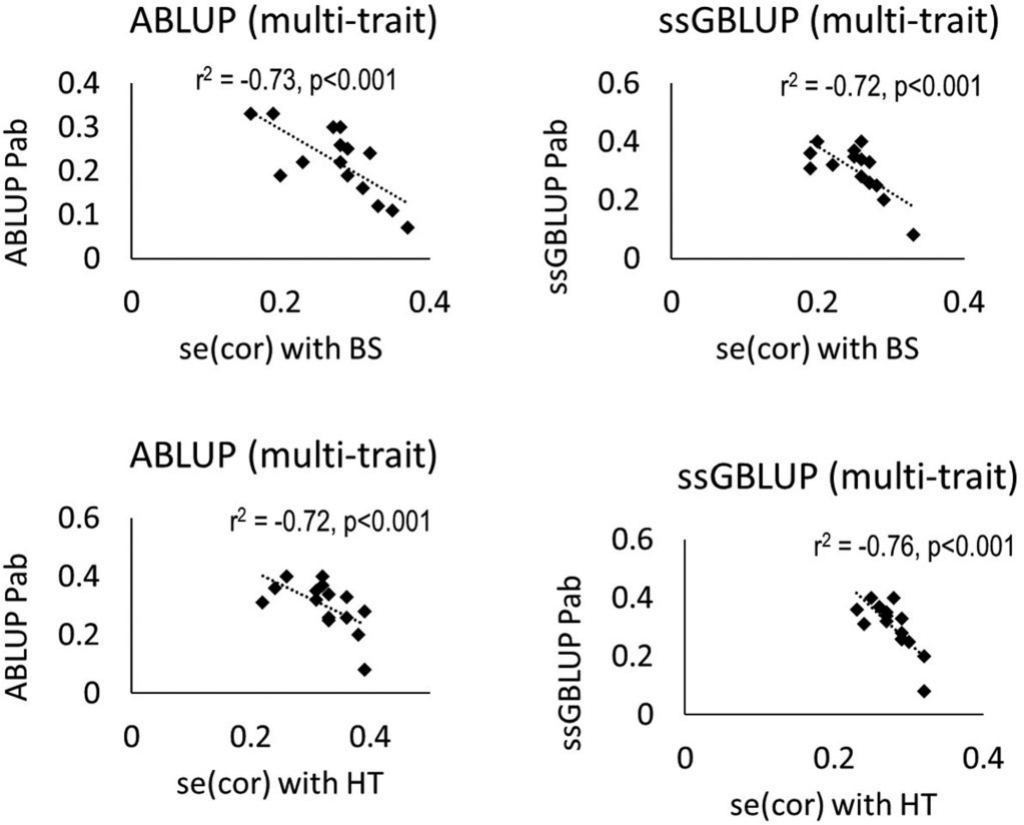
Scatter plots showing regression line and the coefficient of determination (*r*^2^) between predictive ability (Pab) of the bark chemical compounds from multitrait ABLUP and ssGBLUP models and the standard error (SE) of the multitrait ABLUP genetic correlation of the chemical compounds with marsupial bark stripping (BS, above) and height (HT, below). The multitrait models included spatially adjusted marsupial bark stripping, height, and a chemical compound.

In multitrait models, better prediction is expected in the presence of high genetic correlation between the traits. However, there was no significant linear relationship between the predictability of the chemical traits and the genetic correlation of the chemical traits with marsupial bark stripping or height (results not shown). Instead, the SE of the genetic correlation estimates showed a negative linear relationship with predictive ability. This suggests that the precision rather than the magnitude of the correlation (precision interpreted based on SE) has a greater impact on the predictive ability/accuracy. The genetic correlations that were associated with low ABLUP error for estimating the genetic correlation were better predicted than those that had higher genetic correlation SEs (Fig. 1). The effect of data distribution on genomic predictions is presented in Supplementary results (Supplementary File 1).

## Discussion

The heritability estimates were moderate for pedigree-based models. However, significant improvement in the estimates were detected for both single-trait and multitrait genomic models. Various studies have provided evidence that genomic models, using realized relationships based on marker information, lead to a substantial increase in the prediction accuracies for various traits in trees compared to those using pedigree-based relationships (Klápště *et al.* 2014; Ratcliffe *et al.* 2017; Klápště, Dungey, Graham, *et al.* 2020), except for a few cases (Munoz *et al.* 2014). This improvement is related to their ability to accurately trace genetic relatedness, eradicate pedigree errors and the ability to track the genetic variability among full-sibs due to the inheritance of random alleles from the parents—Mendelian sampling variance (Klápště *et al.* 2018). In forest tree breeding, ssGBLUP that combines phenotype, pedigree and genomic information through a single-step genomic evaluation approach has been highlighted as the preferred strategy for evaluation of breeding values where it is not practical to genotype all trees in the large progeny tests used in most forest tree breeding programs (Ratcliffe *et al.* 2017). In this study, while direct evaluations for the impact of GBLUP and ssGBLUP models were made for chemical compounds and height, it was not possible to directly evaluate the impact of GBLUP on marsupial bark stripping estimates since the genotyped trees were not scored for bark stripping. Therefore, the improvement detected in the multitrait ssGBLUP genetic estimates of bark stripping indirectly links to the changes in the accuracy of estimating the genetic estimates of the chemical compound and height that were simultaneously analyzed as response variables in multitrait models. Genotyping of marsupial bark stripped trees is needed to enable a direct assessment of the impact of genomic models to the genetic parameters of bark stripping.

While there are few comparative studies related to herbivory in conifers, one recent study assessed the genomic prediction of resistance against weevil herbivory in spruce (Lenz *et al.* 2020). The authors indicated a 2-fold reduction in the GBLUP heritability compared to pedigree-based prediction for the number of weevil attacks, and also the wood property traits assessed in their study (Chen *et al.* 2018). This is the opposite to what was observed in the current study, which could be related to the nature of SNPs used. The desirable SNPs set will yield optimum genetic estimates if they can capture much information on genetic variation within a defined chromosomal region without introducing redundancy due to extensive linkage disequilibrium (LD) between nearby SNPs (Sun *et al.* 2016). Defining the optimum set of SNPs will, therefore, require knowledge of the patterns of LD across the genome under study, but where that knowledge is lacking, desirable SNPs may possibly increase with the number of SNPs used in a study. In the above study on weevil herbivory for example, Lenz *et al.* (2020) used 4-fold less SNPs than in the current study. Chen *et al.* (2018) and Lee *et al.* (2017) also noted that LD could be reduced by increasing the number of families especially in full-sib populations, so possibly the 40 families (35 parents) used by Lenz *et al.* (2020) were not sufficient to capture the additive genetic variation, although the trees per family were more compared to the current study that used 74 families with up to 6 trees per family. In Norway spruce, predictive ability for several wood traits stabilized as the number of trees within-family reached 6 (Chen *et al.* 2018). Therefore, the optimal parameters to realize the benefits of genomic selection for herbivory may need more research. However, more evidence of the superior performance of genomic models in resistance studies is available from pathosystems in *P. radiata* (Klápště, Meason, *et al.* 2020) and other conifers (Resende *et al.* 2012; Carpenter *et al.* 2018; González-Camacho *et al.* 2018).

Comparing heritability estimates for ABLUP and genomic models showed that the application of genomic-based models significantly improved the narrow-sense heritability estimates of individual chemical compounds compared to the pedigree-based (ABLUP) method. In addition, several chemical traits that did not exhibit statistically significant additive genetic variance with the single-trait ABLUP model did with the GBLUP model. The GBLUP performed better than the ABLUP for most chemical traits except linoleic acid^[59]^, indicating that markers provided additional information. The ssGBLUP, compared to the multitrait ABLUP further improved the estimates highlighting use of correlated information from other traits. However, for some traits like linoleic acid^[59]^, no difference between GBLUP and ssGBLUP were detected, suggesting that the benefit of correlated information in the ssGBLUP is trait specific. Overall, the ssGBLUP and GBLUP should in theory perform well for traits that are under quantitative genetic control. Indeed, there is evidence for their superior performance for different quantitative traits in conifers such as growth and wood traits (Ratcliffe *et al.* 2017; Gamal El-Dien *et al.* 2018; Cappa *et al.* 2019). The genetic control and genomic selection of most chemical compounds have been less studied, and the genetic architecture is less well-known.

Similar to the chemical traits, heritability for height slightly improved with the multitrait ssGBLUP compared with the multitrait ABLUP, consistent with the observations in Norway spruce populations (Chen *et al.* 2018). Our results, however, contrasted with observations in white spruce and Norway spruce (Gamal El-Dien *et al.* 2018; Lenz *et al.* 2020), where multifold reduction in heritability values for height were documented for GBLUP vs ABLUP models. Likewise, in Douglas-fir, Thistlethwaite *et al.* (2019) did not show any relative improvement in heritability estimates in height with genomic models. These contrasting results suggest that improvement in genetic parameter estimation using marker-based approaches relative to pedigree-based methods is contingent upon the trait as well as other factors such as size of the genotyped population, sample size and relatedness of the population, and marker density, which have been variously documented in the literature (Desta and Ortiz 2014; Klápště *et al.* 2018; Momen *et al.* 2018; Stejskal *et al.* 2018). The change in height heritability based on single-trait models is not directly comparable in the present case as the 2 models used different sample sets.

Multitrait models mostly gave higher heritability estimates compared to single-trait models for most of the compounds, which is the norm for most traits (Karaman *et al.* 2020; Lenz *et al.* 2020). Multitrait selection models can improve the accuracy of predictions by taking advantage of the genetic correlations between traits (Calus and Veerkamp 2011). In our case, the benefits of multitrait models were realized since chemical traits had fewer phenotypic records and could be better predicted genetically when coupled with other traits that were extensively assessed (Covarrubias-Pazaran *et al.* 2018). The increase in heritability estimates with multitrait over single-trait models was especially high for traits that had very low heritability values in the single-trait models. However, the magnitude of the genetic correlation from multitrait ABLUP relative to ssGBLUP changed little, consistent with Lenz *et al.* (2020) for the wood and herbivory traits in spruce. Nevertheless, evidence for better performance of the ssGBLUP was derived from the reduction in the SEs of the genetic correlation estimates.

The predictive ability and accuracy (i.e. the ability to predict future phenotypes) were higher with the multitrait ssGBLUP than the multitrait ABLUP. This finding is consistent with some studies in conifers that demonstrated potential improvement of prediction of breeding values with genomic models (Goddard 2009; Hayes *et al.* 2009; Iwata *et al.* 2011; Gamal El-Dien *et al.* 2016; Klápště *et al.* 2018; Stejskal *et al.* 2018; Suontama *et al.* 2018). However, the improvement was only evident in our multitrait models. In multitrait models, studies have indicated that correlations can further improve prediction accuracy when phenotypes are genetically correlated, because measurements on each trait provide information on the genetic values of the other correlated traits (Jia and Jannink 2012). In our study, the ssGBLUP genetic correlations improved relative to ABLUP and consequently the Pab/Pac. Our single-trait models showed no relative advantage of genomic over single-trait pedigree-based models in predictive ability and accuracy, suggesting the influence of genetic correlations between the traits in the improvements noted in the multitrait models (Song *et al.* 2019). This improvement could also reflect the importance of additional individuals in the analysis, especially in that the study showed no linear relationship between predictive ability and genetic correlations involving the other traits in the multitrait model. It could also reflect a reduction in absolute values of the correlations. Instead, predictive ability was strongly negatively correlated with the SE of the genetic correlation estimates. In single-trait models, the lack of improvement in Pab/Pac for the genomic vs ABLUP could be due to various reasons. Firstly, the genotyped reference population may not have been large enough to improve the genomic predictive ability, although it was sufficient to improve the heritability estimates. Few comparative studies exist in conifers, but in animal studies for example, Pab/Pac drastically reduced for genomic compared with ABLUP when the genotyped reference population was small (Lourenco *et al.* 2015; Song *et al.* 2019). In pigs, the change in Pab was insignificant for genomic compared with ABLUP when the genotyped reference population size was <500 (Song *et al.* 2019). Secondly, the parameters selected for the construction of the *H*-matrix, especially *w* (the proportion of the genetic variation not captured by the markers) as well as *α* and *β* (the scaling factors) have a significant impact on the predictive ability. In this study, a low w was selected, which signified that most of the additive genetic variance was captured by the markers, which may not have been the case especially given that the genotyped population was small. The effect of the scaling factor, *w*, has been assessed in various studies and a range of optimum *w* for different traits, up to 0.95 have been established (Oliveira *et al.* 2019; Song *et al.* 2019). Even then, these studies have indicated that *w*, *α*, and *β* can be population and trait specific, such that using the same parameters for different traits may lead to inaccuracy of prediction. Therefore, determining the optimal parameters for these traits is worth investigating.

The GRR modeled increased effects of specific markers, although allowing for such heterogeneity did not improve the Pab/Pac when compared to GBLUP. This contrasts with some studies that have indicated that Pab/Pac could be improved by utilizing the subset of markers with the largest magnitude rather than all markers (Chen *et al.* 2018). Finally, the spread of the data around the mean was of concern in this study since chemical data is often skewed and this distribution has a potential influence on the estimation of the genetic parameters (Kärkkäinen and Sillanpää 2012; Muranty *et al.* 2015). However, skewness (a measure of symmetry) and kurtosis (a measurement about the extremities, i.e. tails, of the distribution of data, which provides an indication of the presence of outliers) had negligible impact on the predictive ability of the pedigree-based or the genomic-based models, although there was a tendency for positively skewed chemical compounds to have higher heritability estimates for all models. Similarly, there was a tendency for compounds with expected kurtosis coefficient (∼3.0) to have higher heritability estimates for all the models (Kim 2013), indicating that the presence of outliers in the data affects the prediction of breeding values more than the symmetry for these models.

In summary, the results here provide the expected response to genomic selection of chemical defense traits in the context of breeding. The results suggest that genomic selection can be useful for improving the accuracy of selecting for chemical phenotypes. This is in addition to other benefits such as reducing the length of the selection cycle, which has been demonstrated in other studies (Li and Dungey 2018). Genomic selection is especially useful for improving selection of traits that exhibit low heritability estimates. Phenotyping has always been a bottleneck in classical breeding programs but the use of high-throughput, low-cost, and labor-saving NIRS enabled the chemotyping of the samples, which provides a promising approach to enable breeders to perform large-scale phenotyping.

## Supplementary Material

jkac245_Supplemental_Figure_S1

jkac245_Supplemental_Figure_S2

jkac245_Supplemental_Figure_S3

jkac245_Supplemental_Figure_S4

jkac245_Supplemental_Material

jkac245_Supplemental_Table_S1

jkac245_Supplemental_Table_S2

## Data Availability

The data underlying this article are available in the article and in its online supplementary material. Genotypic and phenotypic data used in the study is uploaded on Figshare: https://doi.org/10.25387/g3.19070438. Supplemental material is available at G3 online.
