# Supplementary material for: Genomic selection for resistance to mammalian bark stripping and associated chemical compounds in radiata pine
Source: G3 (Bethesda). 2022 Oct 11;12(11):jkac245. doi: 10.1093/g3journal/jkac245 (PMC9635650; doi:10.1093/g3journal/jkac245)
Supplement: jkac245_Supplemental_Figure_S1 [file jkac245_supplemental_figure_s1.pdf]

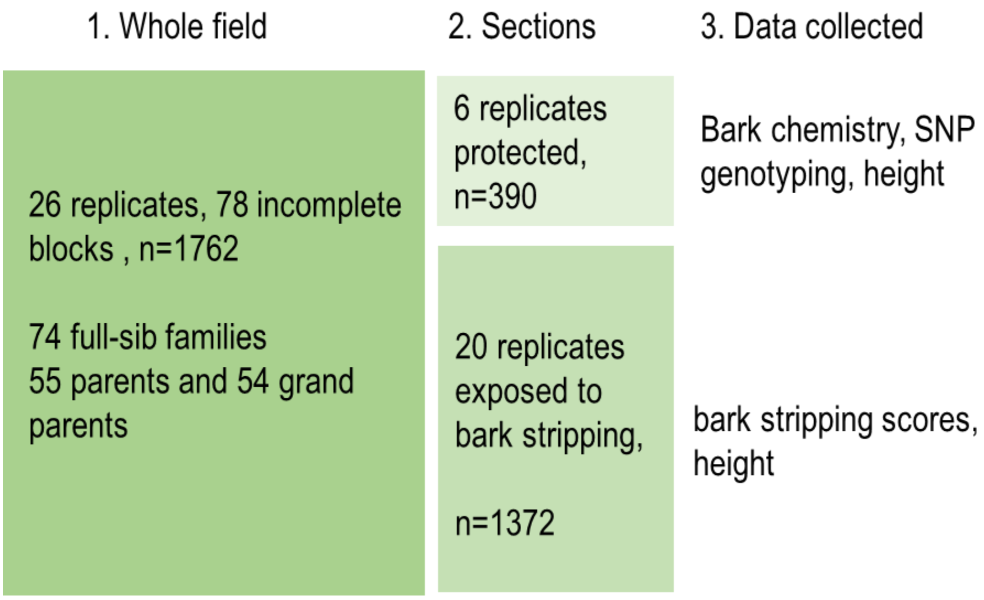

Supplementary Figure S1: Set up of the genetic trial and the data collected from the different sections. The 6 protected replicates were randomly spread throughout the entire field. Height was the only variable assessed in all the 26 replicates, and in the multi-trait models it was the bridging trait between the 20 unprotected and the 6 protected replicates.
