# Supplementary material for: Genomic selection for resistance to mammalian bark stripping and associated chemical compounds in radiata pine
Source: G3 (Bethesda). 2022 Oct 11;12(11):jkac245. doi: 10.1093/g3journal/jkac245 (PMC9635650; doi:10.1093/g3journal/jkac245)
Supplement: jkac245_Supplemental_Figure_S2 [file jkac245_supplemental_figure_s2.pdf]

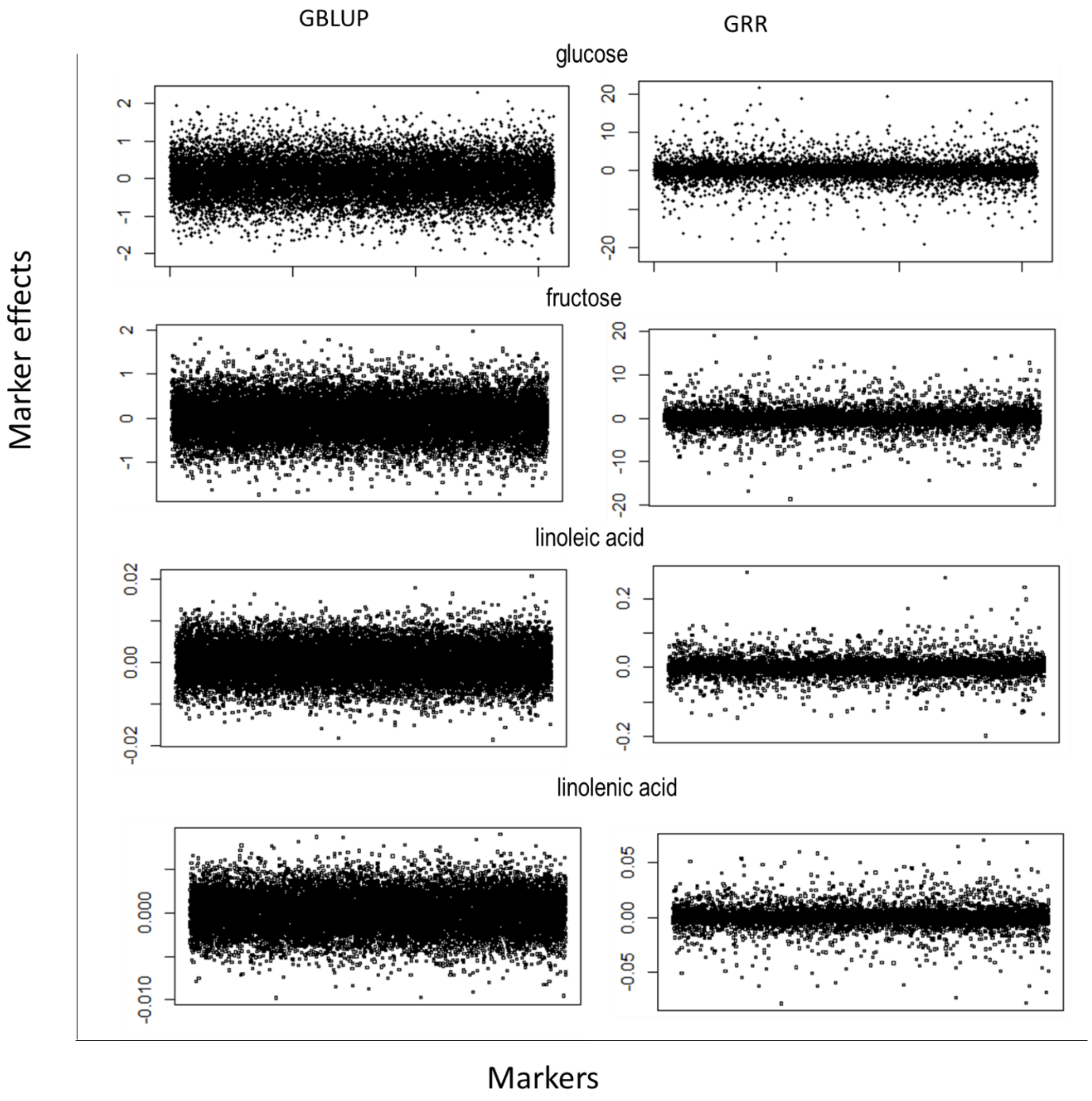

Supplementary Figure S2. Scatter plot showing the estimated effects of each marker for the different chemical traits in the bark of *P. radiata* that significantly correlated with bark stripping estimated using the GBLUP and the GRR.   Note that the GBLUP and GRR effect size scales are different. The change in marker effects is seen on the y-axis. This was done for protected replicates only (n=390)
