# Supplementary material for: Genomic selection for resistance to mammalian bark stripping and associated chemical compounds in radiata pine
Source: G3 (Bethesda). 2022 Oct 11;12(11):jkac245. doi: 10.1093/g3journal/jkac245 (PMC9635650; doi:10.1093/g3journal/jkac245)
Supplement: jkac245_Supplemental_Figure_S3 [file jkac245_supplemental_figure_s3.pdf]

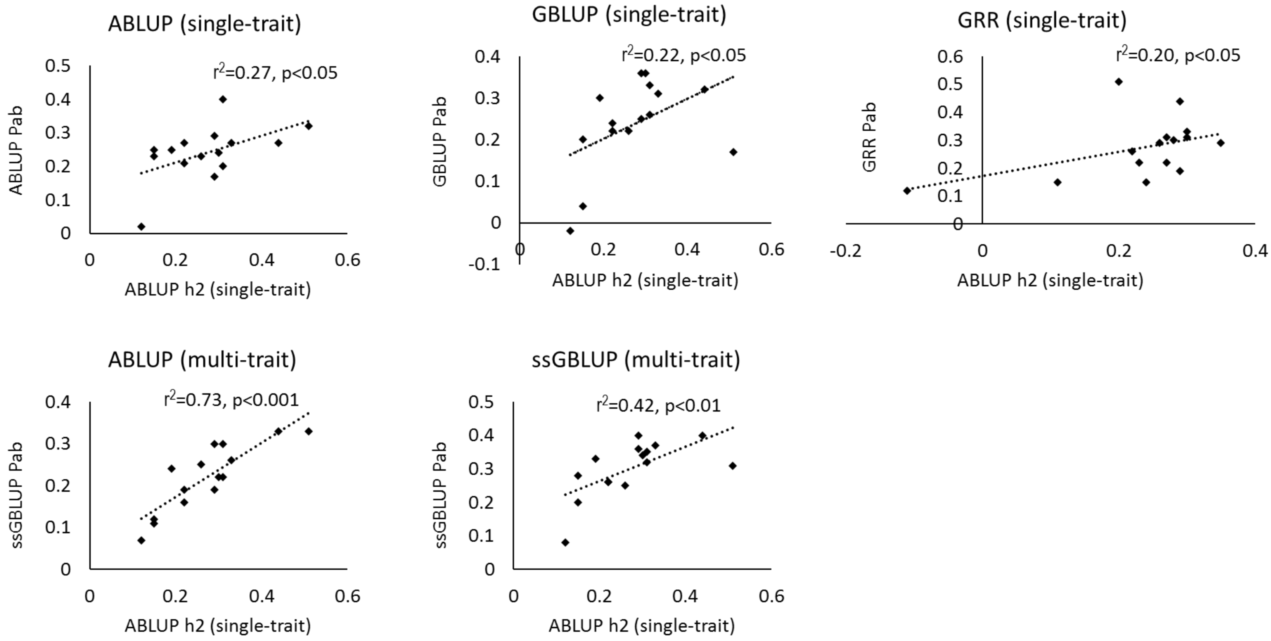


**Supplementary Figure S3:** Scatter plots, regression line and the coefficient of determination ($r^2$) showing the relationship between ABLUP single-trait narrow-sense heritability ($h^2$) and predictive ability (Pab) of single-trait ABLUP and ssGBLUP as well as multi-trait ABLUP and ssGBLUP models. The heritability estimates were derived from single-trait ABLUP models.
