# Supplementary material for: Genomic selection for resistance to mammalian bark stripping and associated chemical compounds in radiata pine
Source: G3 (Bethesda). 2022 Oct 11;12(11):jkac245. doi: 10.1093/g3journal/jkac245 (PMC9635650; doi:10.1093/g3journal/jkac245)
Supplement: jkac245_Supplemental_Figure_S4 [file jkac245_supplemental_figure_s4.docx]

**
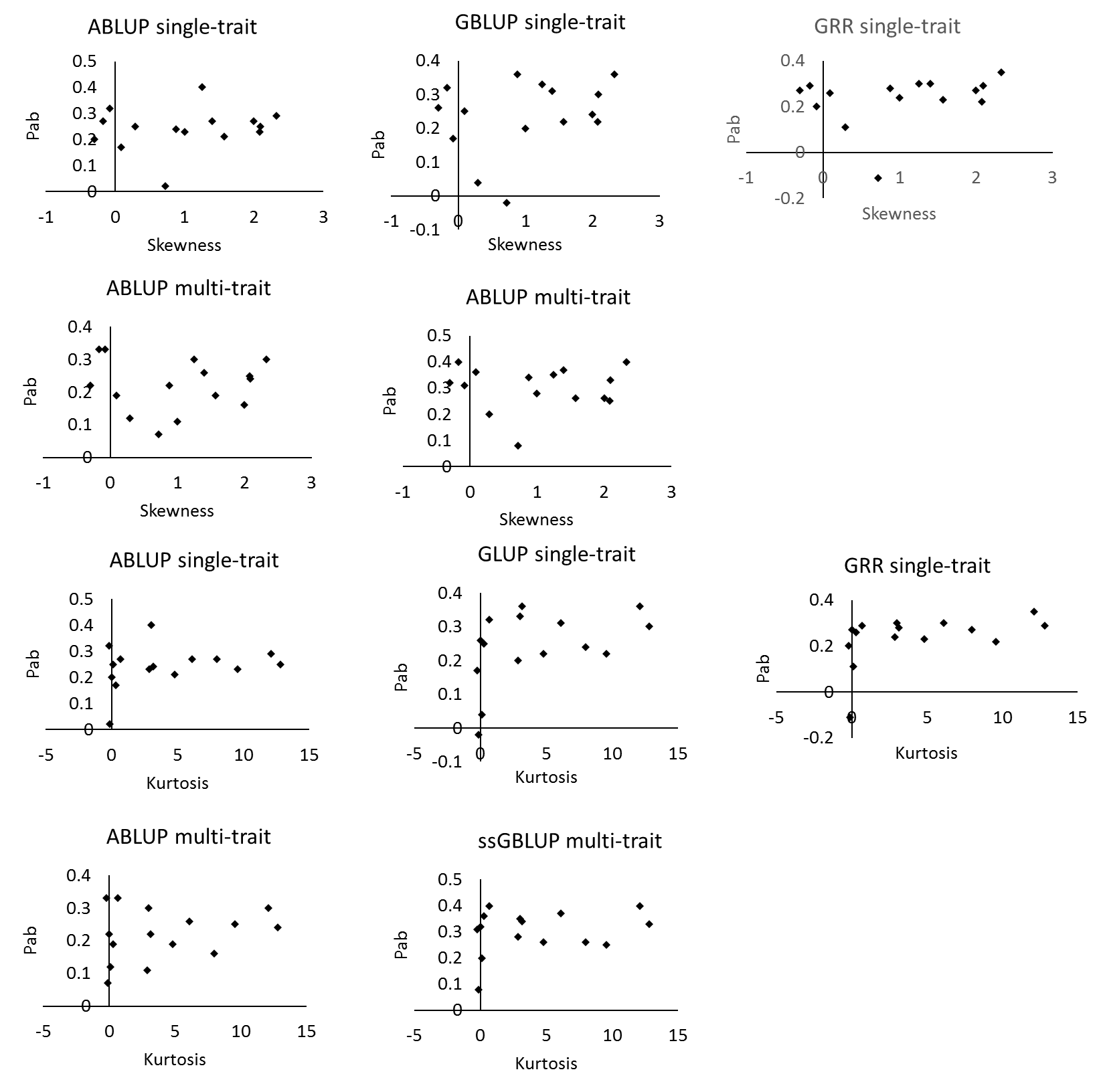
**

**Supplementary Figure S4:** The relationship between predictive ability (Pab) from the single-trait (ABLUP, GBLUP and GRR) and the multi trait (ABLUP and ssGBLUP) models against the skewness (a measure of symmetry) as and kurtosis (a measurement of tails) of the data distribution for the 15 chemical traits. Traits with a normal phenotypic distribution have a skewness of zero and a kurtosis of 3.0, and therefore theoretically highest PA is expected at these points. At the phenotypic level, most compounds exhibited positive skewness (right tail) but the kurtosis values indicate that the tails of the distributions were both under (<3) and over (>3) represented with extreme values compared to that expected from a normal distribution.
