# Supplementary material for: Genomic selection for resistance to mammalian bark stripping and associated chemical compounds in radiata pine
Source: G3 (Bethesda). 2022 Oct 11;12(11):jkac245. doi: 10.1093/g3journal/jkac245 (PMC9635650; doi:10.1093/g3journal/jkac245)
Supplement: jkac245_Supplemental_Table_S1 [file jkac245_supplemental_table_s1.pdf]

Supplementary Table S1: Skewness and kurtosis of bark stripping (n=1372), height (n=1762) and the chemical compounds (n=390) used for estimating heritability, genetic correlation and accuracy of genomic selection. Each chemical compound was given a unique identifier (Id) which follows (Nantongo *et al.* 2021d) for ease of location in the tables.

Id	Compound	Compound group	skewness	kurtosis
	Bark stripping		1.35	0.62
	Height (cm)		-0.35	0.72
1	α-pinene	M	2.08	9.56
4	β-pinene	M	1.40	6.11
5	camphene	M	0.88	3.15
6	citronellal	M	2.09	12.83
18	trans-farnesol	SS	1.00	2.88
20	agathadiol	DG	2.00	7.99
21	agatholal	DG	1.57	4.81
22	copalol	DG	2.33	12.09
23	levopimaral	DG	1.25	3.01
30	dehydroabietic acid	DL	0.72	-0.12
54	fructose	S	-0.30	0.01
55	glucose	S	0.09	0.30
56	inositol	S	0.29	0.13
59	linoleic acid	F	-0.08	-0.21
60	linolenic acid	F	-0.17	0.67
