# Supplementary material for: Genomic selection for resistance to mammalian bark stripping and associated chemical compounds in radiata pine
Source: G3 (Bethesda). 2022 Oct 11;12(11):jkac245. doi: 10.1093/g3journal/jkac245 (PMC9635650; doi:10.1093/g3journal/jkac245)
Supplement: jkac245_Supplemental_Table_S2 [file jkac245_supplemental_table_s2.pdf]

Supplementary Table S2: Genetic correlations and associated standard error (se) of bark stripping [BS] and height [HT] in models with different chemical compounds using ABLUP and ssGBLUP

	Compound		ABLUP cor BS vs HT (se)		ssGBLUP cor BS vs HT	
1	α-pinene	M	0.40	0.29	0.42	0.27
4	β-pinene	M	0.39	0.30	0.43	0.27
5	camphene	M	0.39	0.29	0.41	0.27
6	citronellal	M	0.41	0.29	0.40	0.28
18	trans-farnesol	SS	0.39	0.30	0.43	0.27
20	agathadiol	DG	0.39	0.29	0.41	0.27
21	agatholal	DG	0.39	0.29	0.42	0.27
22	copalol	DG	0.40	0.29	0.41	0.27
23	levopimaral	DG	0.39	0.29	0.42	0.27
30	dehydroabietic acid	DL	0.45	0.29	0.43	0.27
54	fructose	S	0.44	0.29	0.45	0.27
55	glucose	S	0.41	0.29	0.39	0.28
56	inositol	S	0.39	0.29	0.41	0.27
59	linoleic acid	F	0.47	0.27	0.35	0.29
60	linolenic acid	F	0.41	0.28	0.38	0.28
